# Pannexin 1 regulates adipose stromal cell differentiation and fat accumulation

**DOI:** 10.1038/s41598-018-34234-9

**Published:** 2018-11-01

**Authors:** Vanessa R. Lee, Kevin J. Barr, John J. Kelly, Danielle Johnston, Cody F. C. Brown, Kevin P. Robb, Samar Sayedyahossein, Kenneth Huang, Robert Gros, Lauren E. Flynn, Silvia Penuela

**Affiliations:** 1 Department of Anatomy & Cell Biology, Schulich School of Medicine and Dentistry, University of Western Ontario, London, Ontario, N6A5C1 Canada; 2 Biomedical Engineering Graduate Program, University of Western Ontario, London, Ontario, Canada; 3 Departments of Physiology and Pharmacology, and of Medicine, University of Western Ontario, London, Ontario, Canada; 4 Molecular Medicine Research Group Robarts Research Institute, University of Western Ontario, London, Ontario, Canada; 5 Department of Chemical and Biochemical Engineering, University of Western Ontario, London, Ontario, Canada

## Abstract

Pannexin 1 (Panx1) is a channel-forming glycoprotein important in paracrine signaling and cellular development. In this study, we discovered that mice globally lacking Panx1 (KO) have significantly greater total fat mass and reduced lean mass compared to wild type (WT) mice under a normal diet. Despite having higher fat content, Panx1 KO mice on a high fat diet exhibited no differences in weight gain and blood markers of obesity as compared to WT controls, except for an increase in glucose and insulin levels. However, metabolic cage data revealed that these Panx1 KO mice display significantly increased activity levels, higher ambulatory activity, and reduced sleep duration relative to their WT littermates on a high-fat diet. To uncover the cellular mechanism responsible for the increased fat content in the KO, we isolated primary cultures of adipose-derived stromal cells (ASCs) from WT and KO fat pads. In WT ASCs we observed that Panx1 protein levels increase upon induction into an adipogenic lineage. ASCs isolated from Panx1 KO mice proliferate less but demonstrate enhanced adipogenic differentiation with increased intracellular lipid accumulation, glycerol-3-phosphate dehydrogenase (GPDH) enzyme activity, and adipokine secretion, as compared to WT ASCs. This was consistent with the increased adipocyte size and decreased adipocyte numbers observed in subcutaneous fat of the Panx1 KO mice compared to WT. We concluded that Panx1 plays a key role in adipose stromal cells during the early stages of adipogenic proliferation and differentiation, regulating fat accumulation *in vivo*.

## Introduction

The obesity epidemic is the leading cause of global deaths, and according to the World Health Organization approximately 10% of the global population is obese^1^. Obesity accounts for a multitude of comorbidities including cardiovascular disease, type II diabetes, and cancer^2^. Adipocytes, the main cell type responsible for fat accumulation, have the capacity to store large amounts of energy and have a long lifespan, with approximately 10% of adipocytes regenerated annually^3^. In obesity, the innate ability of adipocytes to accumulate lipids causes excess adipose tissue accumulation. Adipose tissue functions as an endocrine organ that plays an active role in inducing associated inflammatory problems^4^. At the onset of obesity, adipose tissue can release many different cytokines and hormones including: leptin, resistin, adiponectin, interleukins (IL-6, IL-1β), tumour necrosis factor alpha (TNF-α), and monocyte chemo-attractant protein-1 (MCP-1)^4^.

Mature adipocytes originate from the expansion and differentiation of a heterogeneous population of multipotent precursor cells and more committed pre-adipocytes, collectively referred to as adipose-derived stromal cells (ASCs)^5^. Adipogenesis is a highly complex process involving dynamic variations in the expression of numerous intracellular and secreted proteins, as well as dramatic changes in cell morphology^6^. Previous research using both the immortalized 3T3-L1 pre-adipocyte cell line^7^ and primary ASC populations^8^ has helped to elucidate the mechanisms of adipogenic differentiation. These cell populations can be induced to differentiate in culture using adipogenic media containing a cocktail of hormones and other factors that stimulate the pathways involved in adipogenesis^9^. Following the induction of differentiation, the cells undergo cell cycle arrest^9,6^, which then triggers a signaling cascade that up-regulates the transcription factors necessary for adipogenesis, including peroxisome proliferator-activated receptor-γ (PPAR-γ) and CCAAT-enhancer binding protein-α (C/EBP-α)^10^. PPAR-γ and C/EBP-α function in concert to help maintain growth arrest^11^ and the differentiative state^12^, and through downstream signaling pathways upregulate the expression of adipogenic genes required for intracellular lipid accumulation and differentiation into mature adipocytes^10^. At early stages, ATP, calcium and other metabolites can regulate the process of adipogenic differentiation from stem cells^13,14^.

Pannexins (Panx) are a family of three channel-forming glycoproteins (Panx1, Panx2 and Panx3) that form channels at the cell surface and intracellular compartments^15,16^. Panx1 has been shown to be ubiquitously expressed in most mammalian organs, while Panx2 (central nervous system^17^, skin, liver^18^), and Panx3 (skin^19^, cartilage^20^ and bone^21^) are more restricted in their expression. Pannexin subunits oligomerize into hexameric functional channels^22^ that allow for the passage of ions and metabolites (<1 kDa) such as ATP^23^ at the cell surface, and calcium in the endoplasmic reticulum^24^.

In adult tissues, Panx1 expression or dysregulation is conducive to the onset or progression of different diseases^25^. However, in early stages of development, Panx1 has been reported to regulate cell proliferation and differentiation in many cell types such as: dermal fibroblasts^26^, keratinocytes^19^, skeletal muscle myoblasts^27^, osteoblasts^28^, and neural progenitor cells^29^. A recent paper showed the importance of Panx1 in lymphatic function and the formation of arteriosclerotic plaque^30^. There is only one report in the literature suggesting that Panx1 is expressed in mature adipocytes and mediates glucose uptake along with insulin sensitivity^31^. However, the role of pannexins in early adipogenic development is currently unknown.

Using a global Panx1 knockout mouse model (Panx1 KO used in this study)^32^, we previously reported that the dorsal skin of the Panx1 KO mice had a thinner dermal area and delayed wound healing capabilities, due to the lack of Panx1 in keratinocytes and dermal fibroblasts^26^. Interestingly, we also noticed that there was a significant increase in the thickness of the hypodermal fat layer of the Panx1 KO that was evident at 4 days of age and persisted into adulthood^26^. Based on this, we hypothesized that Panx1 may regulate adipogenic cell proliferation and differentiation, thus resulting in changes in fat accumulation. In this study, we highlight for the first time the function of Panx1 in early adipocyte development. Panx1 regulates the proliferation and differentiation of murine ASCs, and the germline deletion of Panx1 results in increased fat mass *in vivo*, underlining a role for Panx1 in fat accumulation and obesity.

## Results

### Panx1 KO mice have significantly greater fat mass compared to WT mice

**Figure 1:**
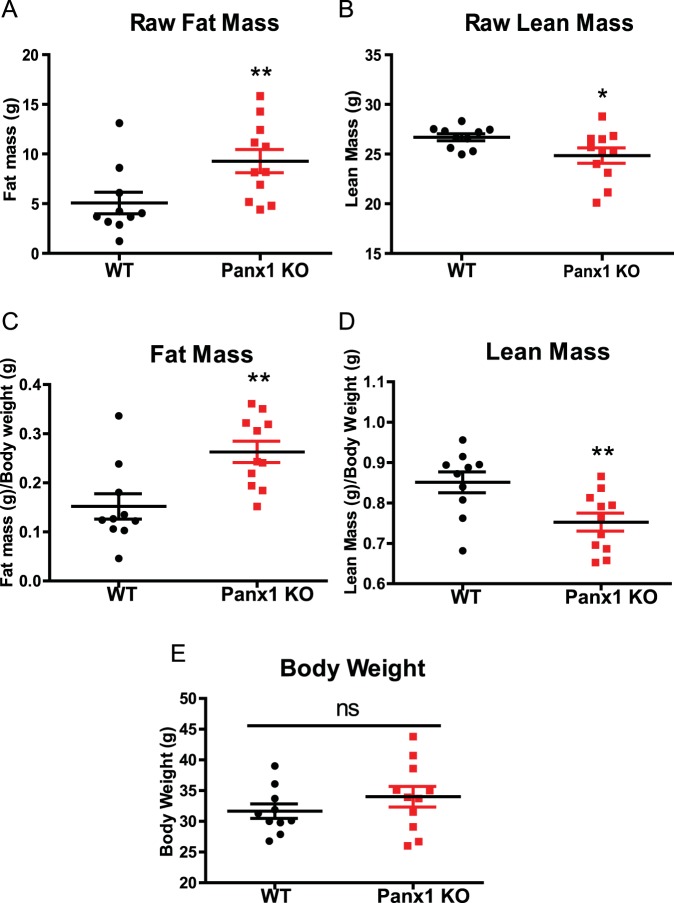
Panx1 KO mice have significantly greater fat mass compared to WT mice. Congenic wildtype (WT) and Panx1 global knockout (KO) mice (12 months-old), were fed ad libitum on a normal chow diet and analyzed with echo-MRI to determine overall body mass composition. Panx1 KO mice had significantly increased raw fat mass compared to WT mice (**A**), as well as overall fat mass (**C**), normalized to body weight. Panx1 KO mice showed a slight decrease in lean mass, when comparing both raw lean mass (**B**) and normalized to body weight (**D**). There was no significant difference in body weight between groups of mice (**E**) WT, N = 10, Panx1 KO, N = 11. ns: not significant, *P < 0.05, **P < 0.01, means ± SEM.

Since we observed previously that Panx1 KO mice had increased subcutaneous fat in the dorsal skin^26^, we set out to evaluate if the overall fat content was increased in the KO. Using the global Panx1 KO mice, described by Qu *et al*.^32^, we backcrossed the mice to C57BL/6 mice until a congenic line of mice was generated (after 10 generations), and assessed their overall body mass composition via echo-MRI. When assessing overall fat mass composition, we observed that Panx1 KO mice had significantly greater fat mass (42% increase) compared to WT mice (WT N = 10, KO N = 11, P < 0.01) (Fig. 1A), and this difference was also significant when the fat mass was normalized to body weight (Fig. 1C). The KO mice had significantly decreased raw lean mass (P < 0.01) (Fig. 1B), and also less lean mass when normalized to body weight (Fig. 1D).

We compared average body weight between WT and Panx1 KO mice and saw no significant differences (WT N = 10, KO N = 11) (Fig. 1E).

### Panx1 KO mice on a HFD show no weight gain differences, but display increased activity

**Figure 2:**
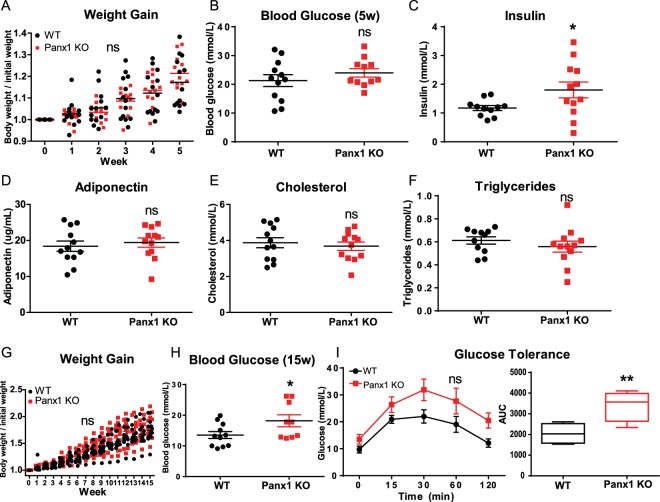
Panx1 KO mice on a HFD show no weight gain differences but slight increases in insulin and glucose levels. WT and Panx1 KO congenic male mice were fed on a high fat diet (60% kcal from fat) for either 5 weeks (**A**–**F**) or 15 weeks (**G**–**I**) starting at 3 months of age. (**A**,**G**) Panx1 KO mice show no difference in body weight increase in either 5- or 15-week HFD. (**B**–**F**) Fasted blood glucose (measured by glucometer), insulin, adiponectin, cholesterol, and triglyceride hormone levels (serum ELISA of WT and Panx1 KO mice fed on a 5-week high fat diet). There were no significant differences between WT or Panx1 KO groups, except for increased insulin levels in the Panx1 KO. (**H**–**I**) Fasted blood glucose collected from WT and Panx1 KO mice fed on a 15-week high fat diet, in addition to a glucose tolerance test where mice were administered 1 g/kg of glucose by intraperitoneal injection and blood glucose was monitored over time. Fasted blood glucose was increased in the KO, and the area under the curve (AUC) for the glucose tolerance test revealed a significant increase in Panx1 KO mice. N = 9–12, means ± SEM. ns: not significant, *P < 0.05, **P < 0.01.

Based on our findings that Panx1 KO mice at baseline had significantly increased fat mass (Fig. 1), we set out to determine whether there would be overt phenotypic effects in the context of a high fat diet. We hypothesized that Panx1 KO mice on a HFD (60% kcal from fat) would show increased weight gain. We placed congenic WT and Panx1 KO mice on a HFD over 5 or 15 weeks and observed no significant differences in body weight increase between WT and KOs (WT N = 13, KO N = 12) (Fig. 2A,G). We looked at blood lipids and various metabolic markers associated with obesity and fat accumulation. At 5 weeks of HFD we did not observe significant changes in glucose measurements but saw a significant increase in insulin levels (Fig. 2B,C), while all other markers (adiponectin, cholesterol, and triglycerides) had no significant differences between groups (Fig. 2D–F). After 15 weeks of HFD, we assessed blood glucose and glucose tolerance, and found significantly higher glucose levels in the Panx1 KO (Fig. 2H). The glucose tolerance test was only significantly different between WT and Panx1 KO mice when comparing the area under the curves (AUC, Fig. 2I).

**Figure 3:**
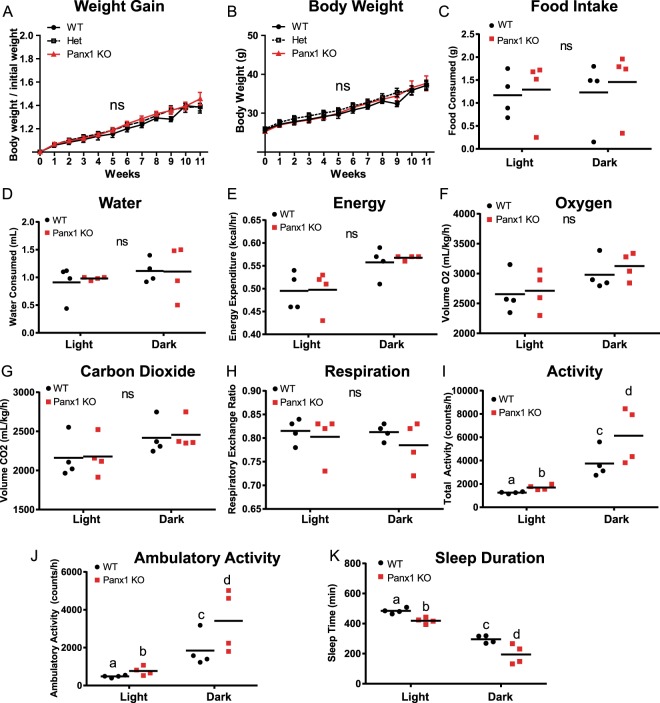
Littermate WT, Het and Panx1 KO mice show no differences in weight increase on a high fat diet, but the Panx1 KO mice exhibit increased activity in metabolic cages. WT, Heterozygous (Het), and Panx1 KO, male littermate mice were fed on a high fat diet for 11 weeks starting at 3 months of age. (**A**–**C**) Panx1 KO mice show no difference in weight gain, body weight, or food consumption. (**C**–**K**) Mice were placed individually in metabolic cages to assess metabolism and activity during their sleep period (light) and during their active period (dark). Panx1 KO mice showed significantly increased total activity (**I**) and ambulatory activity (**J**), with significantly reduced sleep duration (**K**), while all other parameters remained unchanged. (**A** and **B**) WT N = 6, Het N = 13, Panx1 KO N = 13. (**C**–**K**) WT N = 4, Panx1 KO N = 4. Different letters denote significant differences. P < 0.01, means ± SEM.

We also placed littermate (WT, heterozygotes, and Panx1 KO) male mice at 3 months of age on a HFD over 11 weeks and assessed weight and metabolic parameters. We observed that littermates showed no significant differences in body weight increase (WT N = 6, Het N = 13, Panx1 KO N = 13) (Fig. 3A) or raw body weight (Fig. 3B) during the HFD. We placed this cohort of mice individually in metabolic cages (WT N = 4, Panx1 KO N = 4) in order to assess multiple metabolic parameters during their active period (dark) and their sleep period (light) for 24 hours after acclimation. Food and water consumption was monitored and showed no significant differences between WT and Panx1 KO mice (Fig. 3C,D). Further, there were no significant differences in energy expenditure (Fig. 3E), O_2_ volume (Fig. 3F) or CO_2_ volume (Fig. 3G) between the groups of mice. The respiratory exchange ratio (RER) (the rate at which CO_2_ is produced over the rate of O_2_ consumed) was monitored as a measure of how efficiently fuel sources (carbohydrates or fats) were oxidized. However, we did not observe any differences between genotypes (Fig. 3H), suggesting that the diet affected the RER of both WT and Panx1 KO mice to an equal extent. We also measured total activity of the mice and interestingly, the Panx1 KO mice had significantly increased total activity both in the light (26% increase) and dark periods (38% increase) as compared to the WT mice (P < 0.01) (Fig. 3I). Further, we saw that the Panx1 KO mice had significantly greater ambulatory activity both in light (38% increase) and in dark conditions (46% increase) (P < 0.01) (Fig. 3J). Finally, we assessed sleep duration, and found that the Panx1 KO mice were sleeping significantly less than the WT mice during both the light (13% reduction) and dark (34% reduction) periods (P < 0.01) (Fig. 3K). Taken together, the Panx1 KO mice showed no overt weight differences compared to the WT mice, however they showed significantly increased total activity, total ambulatory activity, and they slept significantly less. Therefore, Panx1 KO mice have more total body fat, increased levels of activity, but gained weight at the same rate as WT controls on a HFD. We also tested a separate cohort of WT and KO mice under a normal chow diet in metabolic cages (Supplementary Fig. 1). Although the same trend for increased activity was observed in the Panx1 KO, under a normal diet the differences were not statistically significant, indicating that the activity and sleep phenotypes observed in the Panx1 KO were exacerbated by the HFD.

### Panx1 is expressed in adipose tissue and adipose-derived stromal cells (ASCs)

**Figure 4:**
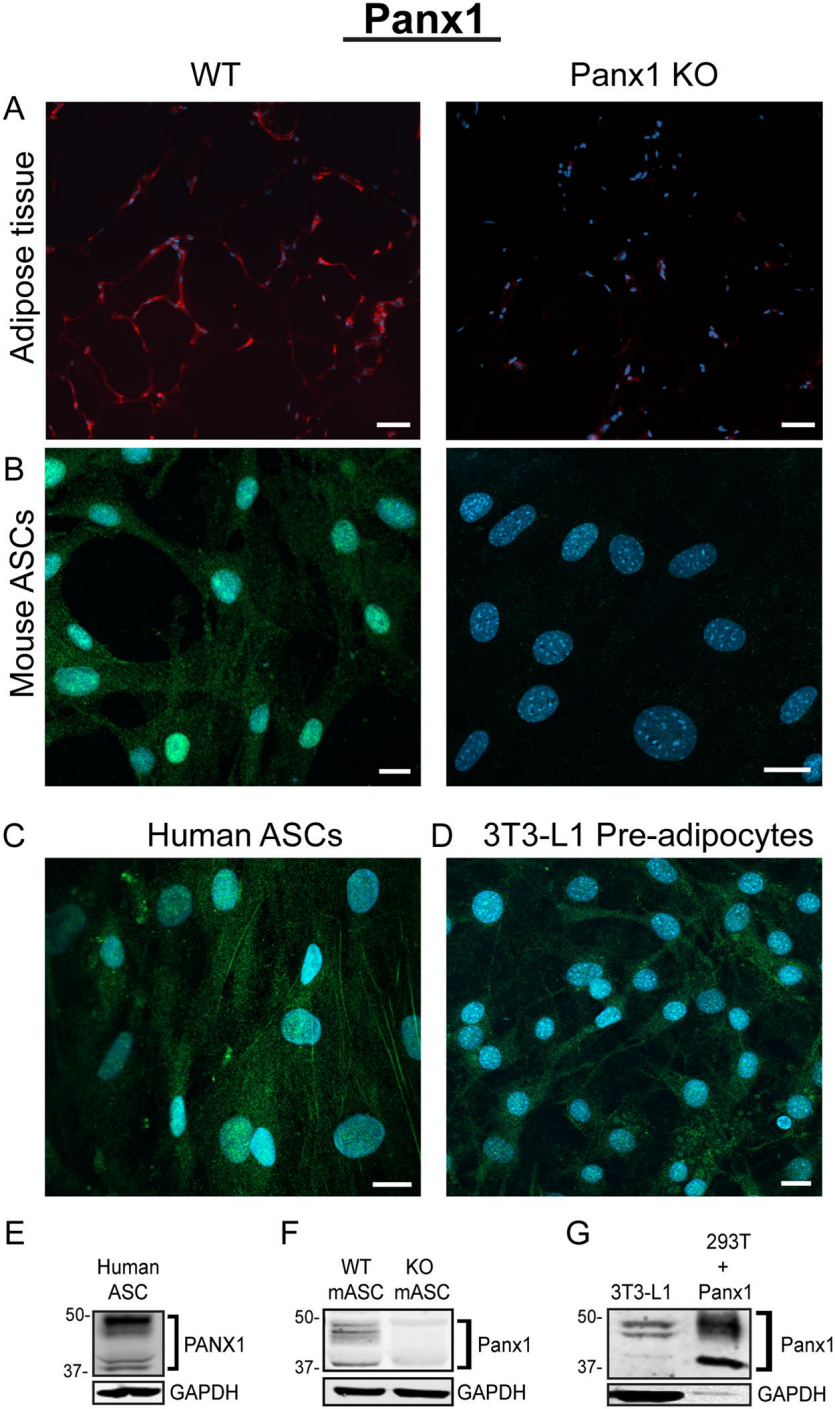
Panx1 is expressed in visceral adipose tissue, mouse and human adipose-derived stromal cells (ASCs), and 3T3-L1 pre-adipocytes. (**A**) Fluorescent micrographs of WT and Panx1 KO adipose tissue from male mice fed on a high fat diet (HFD), depicting Panx1 (red) labeling in the WT and absent in the KO. (**B**) ASCs isolated from adipose tissue of WT and KO mice on HFD and grown in culture, show Panx1 expression (green), that is ablated in the KO cells. (**C**) Human ASCs (hASCs) isolated from female breast adipose tissue express PANX1 (green). (**D**) 3T3-L1 pre-adipocyte cells in culture stained for Panx1 (green). Corresponding Western blots of cell lysates from hASCs (**E**), WT and Panx1 KO mouse ASCs (**F**), mASC), and pre-adipocyte protein lysates plus a positive control of 293 T cells ectopically expressing mouse Panx1 (**G**). Nuclei (blue), scale bars: 44 µm (panel A), 20 µm (**B–D**). GADPH used as loading control. Protein sizes in kDa.

We investigated the cellular mechanism that could be responsible for the increased fat content in the KO mice. As a first step, we labelled white adipose tissue from fat pads of WT mice via immunohistochemistry and observed that Panx1 appeared to be localized either to the plasma membrane of adipocytes in the tissue sections, or in intracellular compartments displaced by the large fat droplets (Fig. 4A). We confirmed that the signal was ablated in the Panx1 KO white adipose tissue (Fig. 4A). Subsequently, we isolated primary ASCs from inguinal fat pads of WT and Panx1 KO mice on HFD (to increase the numbers of viable primary cells obtained). In WT ASCs in culture, we observed that Panx1 was predominantly expressed in the intracellular compartment (Fig. 4B) and Panx1 expression was absent in the ASCs isolated from Panx1 KO mice, as expected. To validate that our findings translated to human cell populations, we also isolated human ASCs derived from two female donors (reduction surgery patients). Immunofluorescence (IF) with anti-human PANX1 antibodies (Fig. 4C) revealed a similar intracellular pattern to that observed with anti-mouse Panx1 antibodies in mouse ASCs and the 3T3-L1 pre-adipocyte cell line (Fig. 4D). When protein lysates from these cell cultures were analyzed by Western blotting, we saw the characteristic multi-banding pattern of Panx1 due to the different glycosylation species in human ASCs (Fig. 4E), WT mouse ASCs (Fig. 4F) and 3T3-L1 cells (Fig. 4G).

### Lack of Panx1 reduces ASC proliferation

**Figure 5:**
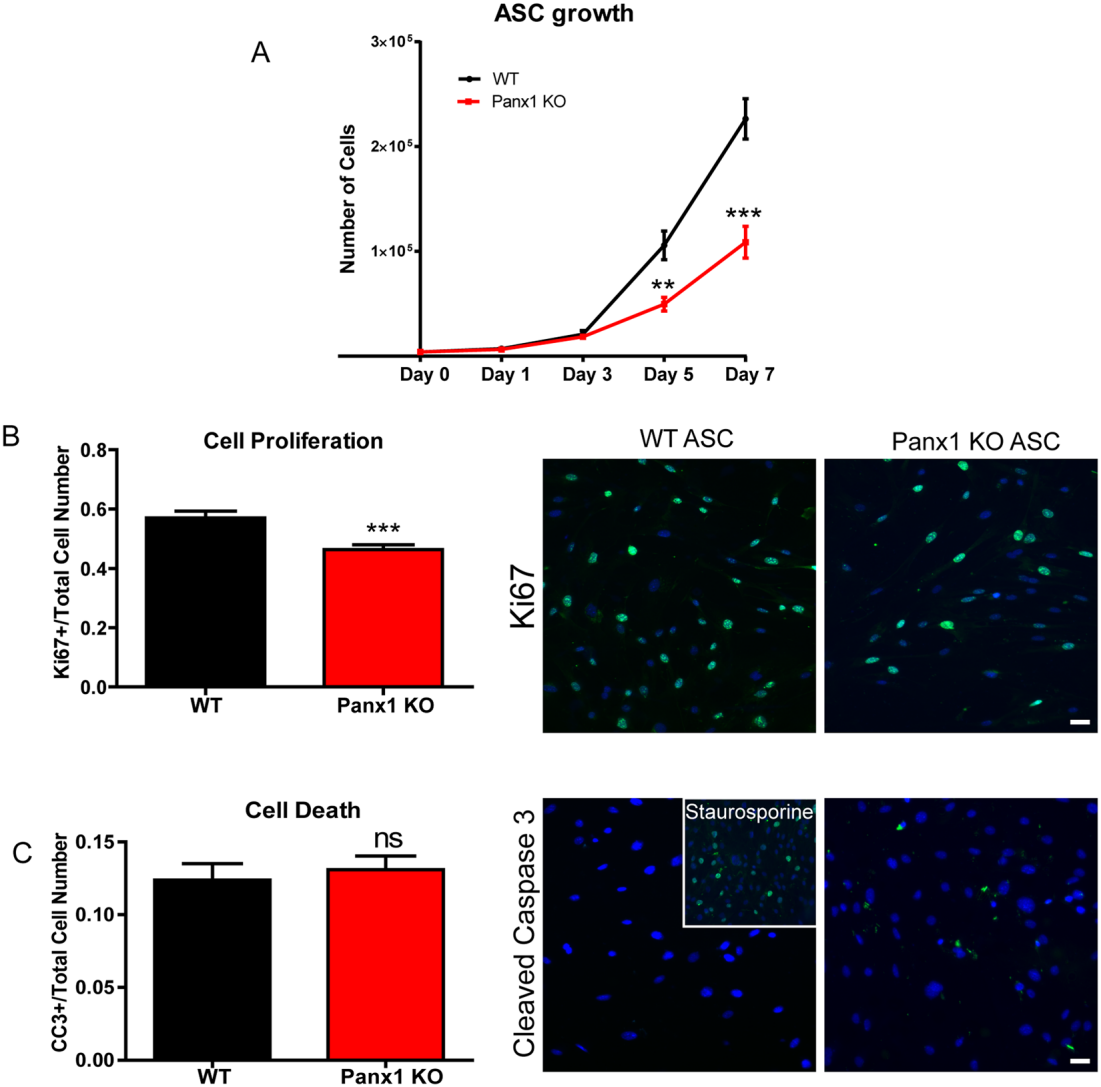
Lack of Panx1 causes a reduction in ASC proliferation. (**A**) Panx1 KO ASCs show significantly decreased growth compared to WT ASCs in a growth curve assay over 7 days. (**B**–**C**) Quantification of (**B**) cell proliferation (Ki67 green, nuclei blue) and (**C**) cell death (cleaved caspase 3 green, nuclei blue), with representative fluorescent micrographs from WT and Panx1 KO ASCs. Panx1 KO ASCs show significantly reduced cell proliferation, and no difference in cell death compared to WT ASCs. Staurosporine induction of cell death in WT ASCs was used as a positive control (insert). Scale bar: 40 µm. A: N = 3, n = 3, (**B** and **C**): N = 3. n = 15, **P < 0.01, ***P < 0.001, means ± SEM.

Since Panx1 is expressed in ASCs (Fig. 4B) and there have been no documented reports of the role of Panx1 in ASCs or in early adipocyte development, we chose to initially assess cell proliferation and viability. We isolated ASCs from the inguinal fat pad of WT and Panx1 KO male littermate mice on a HFD, expanded the cells in culture, and assessed proliferation at passage 2 by systematic cell counts over 7 days. We observed that Panx1 KO ASCs grew significantly slower (Fig. 5A), with an approximately 50% reduction in total cell number compared to WT ASCs at days 5 and 7 in culture (N = 3, n = 3, P < 0.01, P < 0.001). To assess whether this reduction in cell number was an effect of reduced cell proliferation and/or increased cell death, we labeled the cells with the proliferation marker, Ki67 and the apoptosis marker, cleaved caspase 3 by immunocytochemistry. Panx1 KO ASCs proliferated significantly less than WT cells, with a ~20% reduction in Ki67 positive cells (N = 3, n = 15, P < 0.001) (Fig. 5B). When assessing cleaved caspase 3, we saw no significant difference in expression between WT and Panx1 KO ASCs, indicating there was minimal cell apoptosis in both populations (N = 3, n = 15) (Fig. 5C). Therefore, based on these results, Panx1 KO ASCs proliferate significantly slower than WT ASCs, but there was no effect on cell death.

### Adipogenic differentiation is enhanced in Panx1 KO ASCs

**Figure 6:**
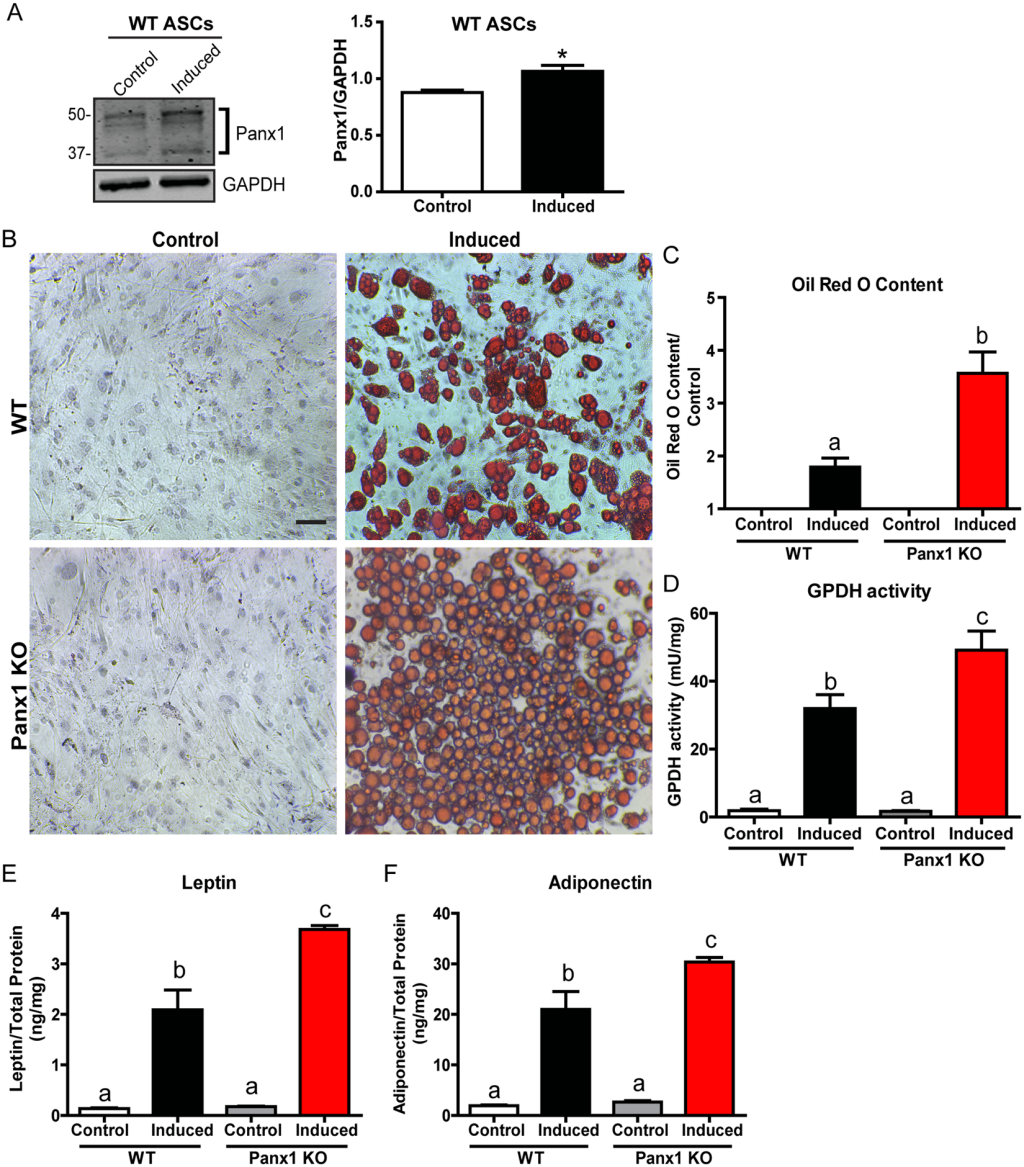
Panx1 expression is increased after adipogenic induction in murine ASCs and lack of Panx1 enhances adipogenic differentiation. ASCs from WT and Panx1 KO mice fed on a HFD were cultured and induced to differentiate into adipocytes for 14 days. (**A**) Western blot and respective quantification of Panx1 expression in WT ASCs showing that expression significantly increases after adipogenic induction. Protein sizes in kDa, GAPDH used as loading control. (**B**) Oil red O staining of WT and Panx1 KO ASCs, control cells (left) and induced cells (right). Scale bar: 100 µm. (**C**) Panx1 KO ASCs had increased oil red O content compared to WT ASCs. (**D**) Panx1 KO ASCs showed significantly higher GPDH activity compared to WT ASCs. (**E** and **F**). ELISAs for leptin and adiponectin performed on conditioned media of ASCs. Panx1 KO ASCs have significantly increased leptin and adiponectin content compared to WT ASCs. (**A** and **D**). N = 3, n = 6, (**E** and **F**). N = 3, n = 9, *P < 0.05, different letters denote significant differences (b: P < 0.01, c: P < 0.05), means ± SEM.

We determined that Panx1 expression was significantly upregulated after 14 days of adipogenic induction (by approximately 18%) in WT ASCs (N = 3, n = 6, P < 0.05) (Fig. 6A). This suggests that Panx1 levels may be low in stem-like progenitor cells (like ASCs), but increases in pre-adipocytes and mature adipocytes throughout adipogenic differentiation. To assess the effect of Panx1 ablation on these stem-like cells, equal numbers of WT and Panx1 KO ASCs were grown in culture in parallel and induced to differentiate in adipogenic differentiation medium over 14 days. When stained with oil red O, we determined that both WT and Panx1 KO ASCs were able to differentiate and accumulate intracellular lipid (Fig. 6B). In contrast, there was no lipid accumulation in control cells maintained in proliferation medium. Comparing the induced populations, we observed that the Panx1 KO ASCs were accumulating more intracellular lipid and had a more mature unilocular morphology, as compared to WT ASCs (Fig. 6B). In fact, the Panx1 KO ASCs differentiated so extensively that the cells were beginning to detach from the plate by 14 days, with a small number of mature adipocytes observed in suspension. To further confirm the enhanced lipid content, we extracted the intracellular oil red O dye from the stained WT and Panx1 KO ASCs and quantified the levels by absorbance spectroscopy, demonstrating that the Panx1 KO induced ASCs had significantly increased levels (~ 45%) as compared to WT induced ASCs (N = 3, n = 6, P < 0.01) (Fig. 6C). To further compare the differentiation response, we measured intracellular glycerol-3 phosphate dehydrogenase (GPDH) activity and observed that the induced Panx1 KO ASCs had significantly higher adipogenic enzyme activity than the induced WT ASCs by ~35% (N = 3, n = 9, P < 0.05) (Fig. 6D). As a final measure of terminal differentiation, we assessed leptin and adiponectin levels in conditioned media from induced and control Panx1 KO and WT ASCs. These adipokines are regarded as late-stage markers of adipogenic differentiation and therefore provide a measure of the maturity of the differentiating cell populations^4,33^. Consistent with the other markers, we saw that Panx1 KO induced ASCs had significantly increased leptin (~40%) and adiponectin levels (~27%) within their conditioned media as compared to WT ASCs (N = 3, n = 9, P < 0.05) (Fig. 6E,F). Therefore, the lack of Panx1 can dysregulate ASC differentiation, enhancing adipogenic differentiation capacity and intracellular lipid accumulation as compared to WT ASCs.

### Lack of Panx1 increases adipocyte hypertrophy and reduces adipocyte numbers in subcutaneous fat *in vivo*

**Figure 7:**
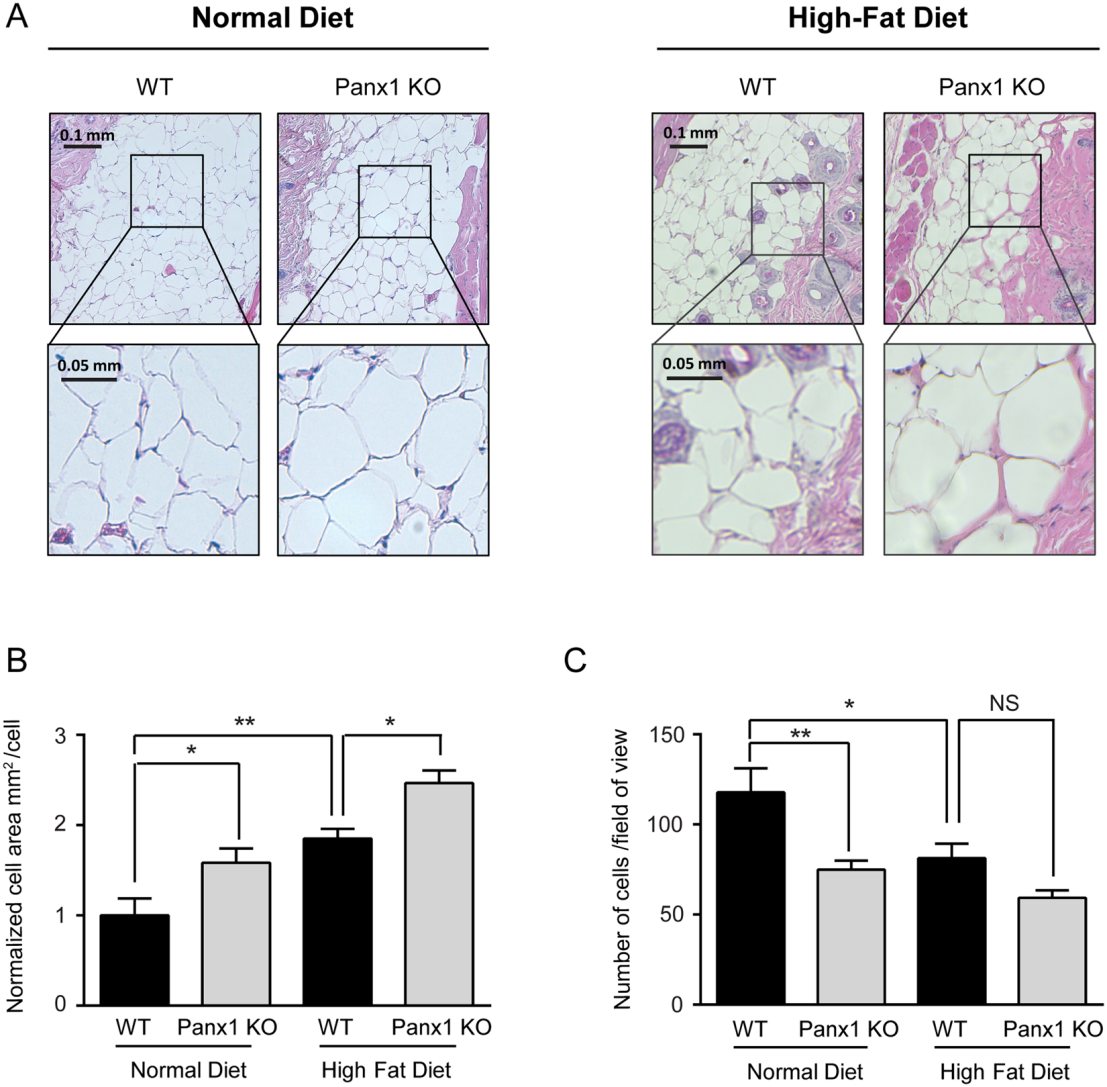
Lack of Panx1 increases cell size and reduces cell number of subcutaneous adipocytes. (**A**) H&E staining of skin from 5-month wild type (WT) or Panx1 knockout (KO) mice on normal chow diet (left panel) or high-fat diet (right Panel). Top rows show lower magnification (scale bar = 0.1 mm) and bottom rows are the insets showing higher magnification of the same image (scale bar = 0.05 mm). (**B**) Graph depicts quantification of adipocyte size in 5-month old wild type (WT) or Panx1 knockout (KO) skin on normal chow or high-fat diet. N = 3 mice per group; Data are normalized to WT on normal chow diet and are expressed as mean + S.E.M from 9 fields per group; * P < 0.05, **P < 0.01, one-way *ANOVA* with Tukey’s multiple comparisons post-test. (**C**) Graph depicts quantification of adipocyte number in each field from 5-month old wild type (WT) or Panx1 knockout (KO) skin on normal chow or high-fat diet. N = 3 mice per group; Data are expressed as mean + S.E.M from 9 fields per group; *P < 0.05, **P < 0.01, one-way ANOVA with Tukey’s multiple comparisons post-test. NS, not significant.

Consistent with our *in vitro* data, we observed a significant increase in adipocyte cell area (an indicator of hypertrophy) in subcutaneous fat pads of Panx1 KO mice under both normal and high fat diet regimes compared to WT (Fig. 7A,B). Adipocyte numbers were significantly decreased in the Panx1 KO fat pads under a normal diet. Under a high fat diet, a similar trend was observed for lower numbers of Panx1 KO adipocytes, but it was not statistically significant (Fig. 7C).

## Discussion

It has been well established that Panx1 has important functions in proliferation and differentiation of many cell types^34,35^, however there have been no reports on its role in adipogenic cell populations. We have shown for the first time that Panx1 regulates the proliferation and differentiation of ASCs into mature adipocytes, and that a germline deletion of Panx1 in ASCs leads to increased adipogenic differentiation and fat accumulation. We have also shown *in vivo* that the global Panx1 KO mouse model has significantly more fat mass than WT controls at baseline. However, the KO mice do not gain more weight under an intense high fat diet, which may be due to their increased activity and decreased sleep relative to their WT counterparts.

The first report on Panx1 being expressed in adipose tissue by Adamson *et al*., 2015 proposed an elegant mechanism in which insulin activates the release of ATP from the Panx1 channels, which in turn causes a signaling cascade indirectly allowing the transport of glucose into the cell^31^. This study also established that blocking Panx1 channels using pharmacological inhibitors significantly reduced glucose uptake in 3T3-L1 pre-adipocyte cells. Additionally, the authors went on to use an adiponectin-inducible Cre recombinase to delete the *Panx1* gene from mature adipocytes, generating an adipocyte-specific Panx1 knockout mouse model (AdipPanx1 KO)^31^. With this model, they found slight diet-induced insulin resistance in the conditional KO, with no changes in body mass composition, metabolic parameters, or activity under a high fat diet^31^. The group also assessed body mass composition in the Panx1 adipose-specific knockout mice on a high fat diet over 12 weeks, and found no significant differences, but observed some trends towards increased circulating blood glucose and increased insulin resistance^31^.

Our study is distinguished from the previous report by the use of the global Panx1 KO mouse with a constitutive deletion of *Panx1* from germline, while the mice in Adamson *et al*. would still express Panx1 at the ASC stage, since the Adiponectin-driven Cre only deletes *Panx1* when the ASCs are already differentiated and express Adiponectin. Our approach enabled the assessment of the role of Panx1 during the earlier stages of adipogenesis, where we observed that the lack of Panx1 in progenitor stem-like cells significantly affected proliferation and differentiation *in vitro*, and fat accumulation *in vivo*. We have demonstrated that the global Panx1 KO mice have significantly greater total fat mass than WT mice, consistent with our previous reports showing that Panx1 KO had a significantly increased area of hypodermal fat under a thinner dorsal skin^26^. We saw no differences in overall body weight, suggesting that the increase in the amount of fat in Panx1 KO mice is subtle. However, we did observe that the overall lean mass was significantly decreased, which could compensate for the increases in fat mass. Moreover, Panx1 is also expressed in bone^36^, muscle^27^ and other organs^37^ that could be altered in this global KO, which may account for the lack of overall differences in body weight. In terms of glucose and insulin, we observed similar trends to those reported by Adamson *et al*.^31^, with slight but significant increases in glucose levels, insulin and area under the curve (AUC) for glucose tolerance in the Panx1 KO. Any differences could be due in part to the higher activity level of the global Panx1 KO mice that may affect glucose levels, compared to the AdipPanx1 KO.

It is possible that the HFD that we chose at 60% kcal from fat (Western HFD chow is only 45% kcal) may have had a saturating effect on the weight phenotype^38^. However, we cannot rule out other effects of the global Panx1 deletion in other organs where the protein is expressed, or the effect of compensation by other channel proteins^26,39^ that may prevent the detection of a more overt weight phenotype. However, the options for a tissue specific Cre-deleter are limited, since the Adiponectin Cre that is the most specific one for fat (used by Adamson *et al*., 2015) only affects cells in late-stage differentiation and mature adipocytes, missing the early stages of adipogenesis where Panx1 plays a key role. In our global KO, overall activity and ambulatory activity were significantly increased in the KO mice, and they also slept significantly less under high-fat diet. In a different global Panx1 KO model^40^, it has also been established that the mice exhibit greater anxiety^41^ and greater motility^42^, thus demonstrating a collective role for Panx1 in increased activity. Recently, the same group that discovered pannexins^43^, reported that another mouse model with a global knockout of Panx1 under normal chow diet, showed similar manifestations in terms of increased waking and decreased slow-wave sleep in the KO mice. They also found that total activity was increased in the Panx1 KOs in both light and dark cycles, consistent with our observations on HFD. However, the increase we observed in activity of Panx1 KO on normal chow diet were not statistically significant (Suppl. Fig. 1), suggesting that those phenotypes may be exacerbated with a HFD. It is well known in the literature that Panx1 is expressed in the brain and central nervous system^28^, and thus a global Panx1 knockout could have a multitude of effects across the body, including at the behavioral and activity level.

Exploring the cellular mechanism behind the increased fat phenotype, we observed predominantly an intracellular localization of Panx1 in all cultured fat cells, making it less likely that Panx1 may act at the cell surface with its canonical ATP release/purinergic signaling function. Immunostaining of the murine adipose tissue shows an apparent cell surface localization in the mature adipocytes. However, it is difficult to visualize the cytoplasm of these cells *in vivo* due to the large accumulation of intracellular lipids. Panx1 is typically seen at the cell surface when ectopically expressed, however it has been reported in the literature that endogenous Panx1 can localize to the intracellular compartments in tissues such as skin^19^, skeletal muscle^27^, canine cardiac myocytes^44^, or in the retina^45^. In primary cells and cell lines, it is common for endogenous Panx1 to localize intracellularly such as in primary dermal fibroblasts^26^. Intracellularly, Panx1 has been proposed to act in the ER as a calcium leak channel^24^. Calcium from the endoplasmic reticulum has been shown to regulate adipocyte differentiation, in studies where increased intracellular calcium inhibited early stages of murine^46^ and human adipocyte differentiation^14^. As such, it is possible that Panx1 in ASCs may function intracellularly as a regulator of calcium and adipogenic differentiation, and the cells that lack Panx1 may be more prone to differentiate. Calcium can also regulate proliferation in mouse pre-adipocytes^47^ so the lack of intracellular Panx1 could potentially alter that balance between proliferation and differentiation and tilt the scale towards differentiation. Consistent with this data, in a previous study assessing Panx1 function in mammary gland development, lactating mice globally lacking Panx1 showed reduced alveolar development and reduced cell proliferation within the mammary glands^48^. In dorsal skin, we showed that primary dermal fibroblasts isolated from the same Panx1 KO model were less responsive to TGF-β stimulation than WT controls, but presented already high levels of α-SMA, a marker of myofibroblast differentiation^26^. These findings suggest that the Panx1 KO fibroblasts may be primed towards differentiation, like our findings with the KO ASC differentiation pattern. Although we propose that calcium signaling regulated by intracellular Panx1 may control proliferation and differentiation of ASCs, we cannot rule out that other signaling pathways like Wnt/MAPK may also be involved in this mechanism. Our published data indicate that knocking down Panx1 in aggressive BL6 melanoma cells results in a substantial reduction of β-catenin abundance, and decreased proliferation^49^. Similarly, in an early stage obesity rat model, a significant reduction in β-catenin levels and increased adipogenesis have been reported to be dependent on Wnt/MAPK-signaling^50^.We propose that intracellular Panx1 may control the differentiation and proliferative capacities of ASCs through modulation of calcium and/or other regulatory pathways such as Wnt signaling. This is the subject of current research efforts by our group and others.

To date, little is known on the role of Panx1 in precursor or stem-like cells throughout differentiation. A recent report found mRNA expression of pannexins in human undifferentiated stem cell lines, with Panx1 showing the highest expression among the three family members^51^. Panx1 is also expressed in postnatal neural stem and progenitor cells, and by either inhibiting or overexpressing Panx1, there were reductions or increases in cell proliferation, respectively^29^. We postulate that Panx1 KO ASCs may be predisposed to the adipogenic lineage, as evidenced by the more homogeneous differentiation response and mature phenotype observed over the 14-day culture period. Further, the lack of Panx1 may also alter the composition of the naturally heterogeneous ASC population. More specifically, there is evidence that multipotent mesenchymal stem cell clones are highly proliferative, whereas unipotent cells exhibit significantly slower growth rates^52^, and lineage commitment leads to reduced proliferation^53^. This is consistent with our results where Panx1 KO ASCs have significantly reduced proliferation, which may indicate further commitment towards the adipogenic lineage as compared to WT ASCs. *In vivo*, these changes in differentiation and proliferation of the ASCs may lead to the increased hypertrophy of the mature adipocytes that we observed in Panx1 KO subcutaneous fat, with a concomitant reduction in adipocyte cell numbers in these fat tissues.

In summary, we have shown for the first time that Panx1 participates in the balance between proliferation and differentiation of precursor adipogenic cells at early stages of development, regulating fat accumulation. We have demonstrated that Panx1 KO mice have higher fat content but show no overt weight gain increase on a high fat diet, potentially due to their increased activity and sleep alterations. Taken together, we have identified Panx1 as a novel regulator of ASC proliferation and adipogenic differentiation, and consequently, a key component of the regulation of fat accumulation, representing a potential new target for obesity intervention.

## Materials and Methods

### Animals and ethics

Experiments performed on animals were approved by the Animal Care Committee of the University Council on Animal Care at the University of Western Ontario, London ON, Canada (UWO # 2015-035), and were performed in accordance with relevant guidelines and regulations. Panx1 KO mice were a kind gift from Genentech Inc. (San Francisco, CA) and were previously described^25,26,32^. These mice were backcrossed with the WT mice: C57BL/6 N strain mice from Charles River Canada (Saint-Constant, PQ) until a congenic line was obtained (minimum of 10 backcrossed generations). Human samples were obtained from breast reduction surgeries from patients after informed consent was obtained, at the London Health Research Centre, according to the guidelines and regulations approved by The University of Western Ontario Research Ethics Board for Health Sciences Research Involving Human Subjects (HSREB) (ethics protocol REB# 105426), in accordance to the Tri-Council policy statement: Ethical Conduct of Research Involving Humans and the Health Canada/ICH Good Clinical Practice Practices: Consolidated guidelines and the applicable laws and regulations of Ontario.

### High fat diet

Congenic and littermate WT and Panx1 KO male mice were placed on a high fat diet (HFD, 60% kcal from fat, Test Diet 58Y1) for up to 15 weeks and fed *ad libitum*. Mice were 3 months old at the start of the experiments and initially fed normal chow diet (6.2% kcal from fat).

### Body mass composition

Fat and lean mass composition of 12 month-old Panx1 knockout male mice, were measured using quantitative magnetic resonance (echo-MRI) analysis with an echo magnetic resonance imaging mobile unit (Avian Facility of Advanced Research, University of Western Ontario, London, ON, Canada) as described by^54^, with the modification of placing live mice in the apparatus and measuring on the ‘small animal’ setting omitting water content. Measurements were taken in duplicate to verify the results.

### Immunofluorescence

White adipose tissue (WAT) was isolated from WT and Panx1 KO mice after a 15-week high fat diet, by incising the abdomen of the mouse and excising all visible fat. Adipose tissue was fixed in 10% neutral buffered formalin overnight, processed at the Robarts Research Pathology facility and embedded in paraffin. Paraffin sections were taken at 6 µm thickness, sections were deparaffinized and immuno-labeled with Panx1-CT primary antibody (stock 1 μg/μl) in a 1:500 dilution as described previously by Penuela *et al*.^37^. For proliferation studies, cells were labelled with active cell cycle phase specific marker Ki-67 in a 1:1000 dilution (Abcam, Cambridge, UK), and for cell death assays cells were labelled with CellEvent (Thermo Fisher Scientific) Caspase-3 Green Detection Reagent following manufacturer’s protocols, and counterstained with Hoechst nuclei stain in a 1:1000 dilution, as described previously^26^.

### Histological staining and subcutaneous adipocyte measurements

Dorsal skin samples from adult male mice (6-months old) on either normal or high-fat diet, were fixed in 10% neutral buffered formalin and subsequently embedded in paraffin. Sections (5 µm) were deparaffinized in xylene, rehydrated in graded alcohols, and washed in PBS. Parallel tissue sections were stained with hematoxylin/eosin. Images were collected using a Leica DM IRE2 inverted epifluorescence microscope. Measurement of adipocyte cellular size (area) and number of measured adipocytes was performed using the analytical software ImageJ (v.1.50i, National Institute of Health, USA). At least three tissue sections from each mouse were analyzed and individual adipocytes with complete boundaries were selected for quantification and counting. Data analysis was performed using GraphPad Prism® Ver. 6.07 (GraphPad Software, Inc.). One-way *ANOVA* test and Tukey’s multiple comparisons post-test were used to analyze the data. *p* < 0.05 was considered significant.

### 3T3-L1 cell culture

Mouse embryonic fibroblast pre-adipocyte (3T3-L1) cells (ATCC) were grown in Dulbecco’s Modified Eagle’s Medium (DMEM) with 4.5 g/L glucose, 1% Pen-Strep, and 10% calf serum (Thermo Fisher Scientific) and cells below passage 10 were included in the studies.

### Adipose-derived stromal cell isolation

ASCs were isolated as previously described by Yu *et al*.^55^ from WT and Panx1 KO littermate male mice fed on the 11-week HFD, with the modification of isolating cells from the inguinal adipose depot and cells were filtered through a 100 μm filter to remove debris prior to cell seeding. Fat from up to three mice was pooled together for each separate isolation. Cells were seeded at high density (80 000 cells/cm^2^) and rinsed 24 hours after isolation with sterile PBS, and passaged when confluent (approximately 7 days). ASCs were grown in DMEM: Ham’s F-12 (Sigma Aldrich), supplemented with 10% fetal bovine serum and 1% Pen-Strep and growth medium was changed every 2 days. ASCs used for assays were grown to Passage 2. Human ASCs were isolated from two female breast adipose tissue donors as described previously^56^.

### Western Blotting

Western blots of protein lysates (primary cells and cell lines) were conducted as described previously by Penuela *et al*.^37^. Cell lysates were collected with a Triton-based extraction buffer [1% Triton X-100, 150 mM NaCl, 10 mM Tris, 1 mM EDTA, 1 mM EGTA, 0.5% Np-40, 100 mM NaF, 100 mM sodium orthovanadate, proteinase inhibitor mini-EDTA tablet (Roche-Applied Science, Laval, QC)] and subsequently run on a Western blot, then probed using the Panx1-CT antibody at 1:5000 dilution (0.2 μg/ml), or anti-hPANX1 (412–426) antibody^57^ at 1:1000 dilution (1.0 μg/ml), with the modification of using 50 μg of protein for all blots^37^. Human embryonic kidney (293 T) cells ectopically expressing mouse Panx1 were used as a positive control for all mouse blots as described by Penuela *et al*.^36^. Loading control used was GAPDH (Millipore, Billerica, MA).

### Proliferation and differentiation assays

ASCs from WT and Panx1 KO mice were plated for proliferation studies in 12-well plates at a seeding density of 10 000 cells/cm^2^. Cell counts were measured in triplicate every other day up until day 7 using trypan blue (1:1) and a Countess automated cell counter (Thermo fisher Scientific). For differentiation assays, ASCs were plated in 6-well plates at a seeding density of 30 000 cell/cm^2^. Adipogenic media was prepared as previously described^55^, with the modifications of substituting 1 µg/mL troglitazone and 0.25 mM isobutylmethylxanthine (IBMX) (instead of 0.5 mM) (Sigma Aldrich) for days 1–3. After day 3, a modified adipogenic media was made, lacking troglitazone and IBMX. Modified adipogenic media was changed every other day until day 14 when the differentiation assays were performed. GPDH enzyme activity was measured using the GPDH Activity Measurement Kit (Kamiya Biomedical Corporation, Seattle, WA, USA) following the manufacturer’s protocols and as described previously^58^. GPDH activity was normalized to total intracellular protein content measured using the Bio-Rad Protein Assay.

### Oil Red O staining

For visual inspection of differentiating cells, plates were stained with oil red O (Sigma Aldrich) after 14 days of differentiation as previously described by Flynn *et al*.^58^, with the modification of an 8-minute oil red O incubation, followed by hematoxylin counterstaining for 2 minutes. Staining was visualized on a bright field microscope and images were recorded in 4 randomly selected areas from each well. To quantify oil red O content, the intracellular dye was extracted by incubating the stained cells in 100% isopropanol at room temperature for 15 minutes. The absorbance of the dye extract was measured at 492 nm using a CLARIOstar (BMG Labtech) plate reader to compare the relative intracellular lipid content between groups.

### ELISA assays

Conditioned media from induced ASCs and 3T3-L1 wells, as well as non-induced controls were collected at 14-days post-induction, where the media had been conditioned for 48 hours prior to analysis. Leptin and adiponectin content in the media was measured by ELISA following the manufacturer’s protocols (Crystal Chem Inc., IL, USA). Total leptin and adiponectin levels in the supernatants were normalized to total intracellular protein content measured using the Bio-Rad Protein Assay. Additionally, for the *in vivo* studies, ELISAs (ALPCO, NH, USA) were performed following the manufacturer’s protocols for insulin and adiponectin, while cholesterol was assessed by CHOD-PAP kit (Roche Diagnostics, Indianapolis, IN) and triglyceride analysis was conducted by Triglycerol/Glycerol kit (Roche Diagnostics, Indianapolis, IN) following manufacturer’s protocols. Analyses for the *in vivo* studies were conducted using serum collected from congenic mice fed on a 5-week HFD.

### Metabolic analysis

Blood glucose analysis was conducted at the time of termination, while glucose tolerance testing was conducted one week prior to termination. Male congenic mice that were placed on a 15-week HFD were fasted 4 hours prior to testing. Fasted blood glucose was measured via a glucometer (OneTouch Ultra) and glucose tolerance testing was conducted by administration of 1 g/kg of glucose by intraperitoneal injection and blood glucose was monitored at 15, 30, 60, and 120 minutes. Metabolic analysis was assessed using the Comprehensive Lab Animal Monitoring System (CLAMS) with the Oxymax software (Columbus Instruments, Columbus, OH, USA) at the Robarts Research Institute. Mice were individually caged and acclimated for 24 hours prior to measurement of food consumption, water consumption, energy expenditure, volume of oxygen (VO2) and carbon dioxide (VCO2), respiratory exchange ratio (RER), total activity, total ambulatory activity, and sleep duration, as described previously^59^.

### Statistical analysis

Statistical analyses were performed using Graph Pad Prism (GraphPad, San Diego, CA). Outliers were removed from data sets using outlier tests from GraphPad Prism Ver 6.07. Student’s (unpaired) t-tests or ANOVA were performed with Tukey’s *post hoc* comparisons. Data are presented as mean ± SEM. N values in *in vitro* ASC assays represent pooled cells from up to 3 mice, whereas, N values in *in vivo* experiments represent individual mice. Technical replicates indicated by n.

## Electronic supplementary material

Supplementary Figure 1

## Data Availability

Raw data and analysis files can be made available upon request. No data sets have been submitted elsewhere.
