# Supplementary material for: Pannexin 1 regulates adipose stromal cell differentiation and fat accumulation
Source: Sci Rep. 2018 Nov 1;8:16166. doi: 10.1038/s41598-018-34234-9 (PMC6212408; doi:10.1038/s41598-018-34234-9)
Supplement: Supplementary file 1 — Supplementary Figure 1 [file 41598_2018_34234_MOESM1_ESM.pdf]

Pannexin 1 regulates adipose stromal cell differentiation and fat accumulation

Vanessa R. Lee[1], Kevin J. Barr[1], John J. Kelly[1], Danielle Johnston[1], Cody F.C. Brown[1], Kevin P. Robb[2], Samar Sayedyahossein[1], Kenneth Huang[1], Robert Gros[3, 4], Lauren E. Flynn[1, 5], Silvia Penuela.[1*]

[1] Departments of Anatomy & Cell Biology, [2]Biomedical Engineering Graduate Program, [3]Physiology and Pharmacology, [4]Robarts Research Institute, Schulich School of Medicine & Dentistry, [5]Chemical and Biochemical Engineering, University of Western Ontario, London, Ontario, N6A5C1, Canada.

[*]corresponding and senior author: spenuela@uwo.ca

Metabolic Cage Data - Normal Diet

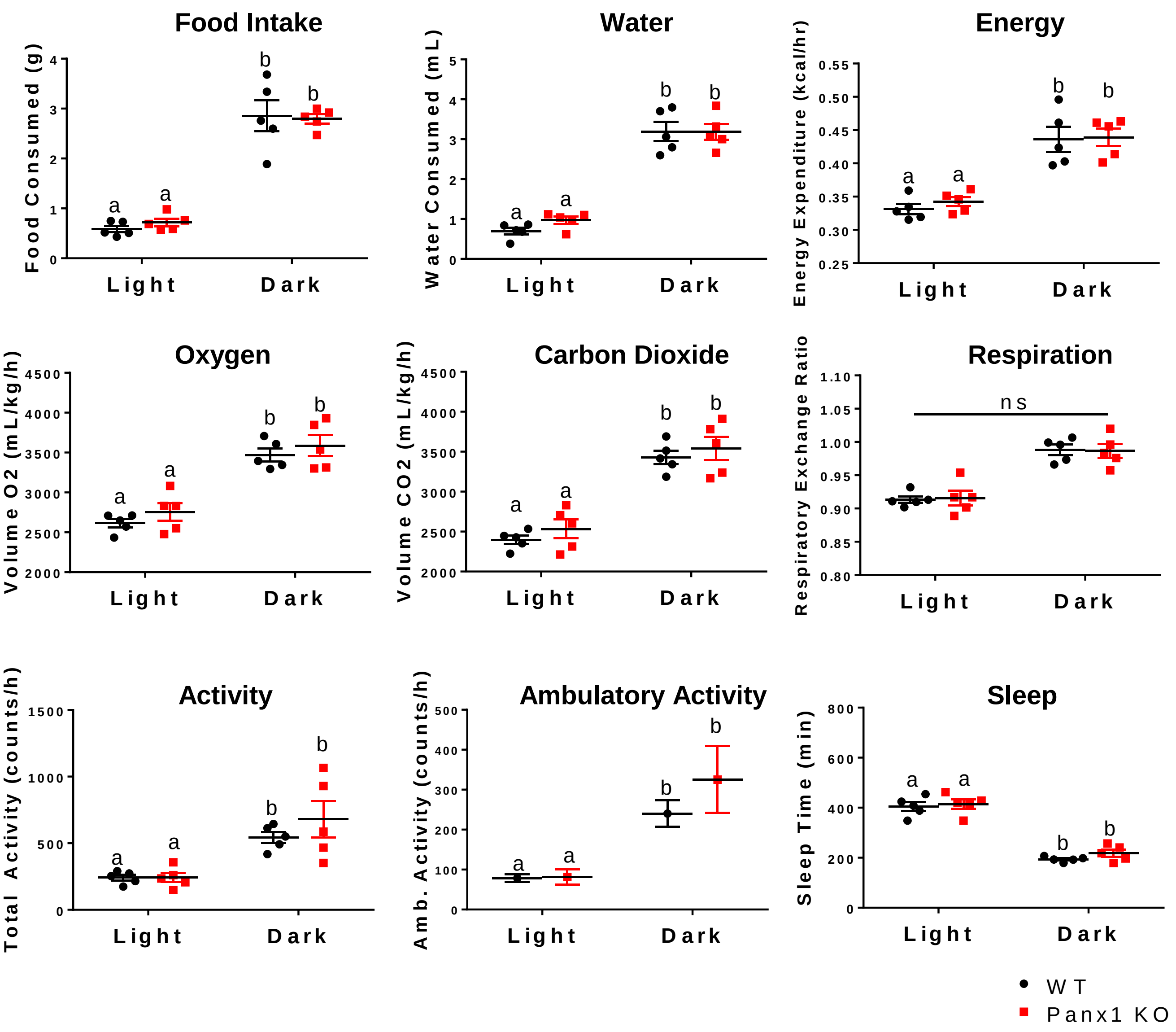

Supplementary Figure 1. Metabolic cage analyses on normal chow diet. WT and Panx1 KO, male littermate mice were fed on a regular chow diet and were placed individually in metabolic cages to assess metabolism and activity during their sleep period (light) and during their active period (dark). Panx1 KO mice show no difference in food consumption, water intake, energy expenditure, oxygen, carbon dioxide, or respiration. Panx1 KO mice showed a slight but not-significant increase in total activity and ambulatory activity (dark period) while all other parameters remained unchanged. N=5, One-way ANOVA, $P<0.01$, means ± SEM. Different letters denote significant differences.
